# Substance Use Affects Type 1 Diabetes Pancreas Pathology: Implications for Future Studies

**DOI:** 10.3389/fendo.2021.778912

**Published:** 2021-11-29

**Authors:** Brittany S. Bruggeman, Martha Campbell-Thompson, Stephanie L. Filipp, Matthew J. Gurka, Mark A. Atkinson, Desmond A. Schatz, Laura M. Jacobsen

**Affiliations:** ^1^ Department of Pediatrics, Division of Endocrinology, University of Florida, Gainesville, FL, United States; ^2^ Diabetes Institute, University of Florida, Gainesville, FL, United States; ^3^ Department of Pathology, Immunology and Laboratory Medicine, University of Florida, Gainesville, FL, United States; ^4^ Department of Health Outcomes and Biomedical Informatics, University of Florida, Gainesville, FL, United States

**Keywords:** islet amyloid polypeptide, islets of langerhans, pancreatitis, pathology, substance-related disorders, tissue donors, type 1 diabetes mellitus, exocrine pancreas

## Abstract

Access to human pancreas samples from organ donors has greatly advanced our understanding of type 1 diabetes pathogenesis; however, previous studies have shown that donors have a high rate of substance use, and its impact on pancreatic histopathology in this disease is not well described. One-hundred-thirty-one type 1 diabetes and 111 control organ donor pancreata from persons 12-89 years of age (mean 29.8 ± 15.5 years) within the Network for Pancreatic Organ donors with Diabetes (nPOD) were examined for insulin positivity, insulitis, amyloid staining, acute and chronic pancreatitis, and chronic exocrine changes (acinar atrophy, fibrosis, fatty infiltration, or periductal fibrosis); findings were compared by history of substance use. A secondary analysis compared exocrine pancreatic histopathologic findings in type 1 diabetes versus control organ donors regardless of substance use history. We observed a high but congruent rate of substance use in type 1 diabetes and control organ donors (66.4% and 64% respectively). Among donors with type 1 diabetes (but not controls), islet amyloid (OR 9.96 [1.22, 81.29]) and acute pancreatitis (OR 3.2 [1.06, 9.63]) were more common in alcohol users while chronic exocrine changes (OR 8.86 [1.13, 69.31]) were more common in cocaine users. Substance use impacted the pancreata of donors with type 1 diabetes more than controls. Overall, despite similar rates of substance use, acute pancreatitis (15.3% versus 4.5%, p=0.0061), chronic pancreatitis (29.8% versus 9.9%, p=0.0001), and chronic exocrine changes (73.3% versus 36.9%, p<0.0001) were more common in type 1 diabetes donors than controls. Alcohol and/or cocaine use in type 1 diabetes organ donors increases exocrine pancreas pathology and islet amyloid deposition but does not affect insulitis or insulin positivity. Exocrine pathology in type 1 diabetes donors is common, and further study of the pathophysiology of these changes is needed.

## 1 Introduction

Human pancreata from the Network for Pancreatic Organ donors with Diabetes (nPOD) program have been used extensively to study insulitis and many other islet related features in type 1 diabetes (1). Beyond this, there has also been increasing interest in the study of exocrine pancreatic changes in those with this disease (2, 3).

There is a high rate of substance use, including alcohol, marijuana, and cocaine, in nPOD donors with type 1 diabetes (4). It is unknown whether this high rate is unique to donors with this disease or similar across all study groups. Alcohol, cocaine, marijuana, and methamphetamine use have been linked to diabetic ketoacidosis (DKA), possibly due to insulin omission while using these substances versus a direct impact on insulin secretion and glucose metabolism (5–9). Additionally, very heavy alcohol consumption and smoking are well known risk factors for chronic pancreatitis, with alcohol, cocaine, and opioid use linked to presentations of acute pancreatitis and altered insulin secretion profiles (10–14). Studies have conflicted when evaluating the role of cannabis in pancreatic inflammation, beta cell function, and acute pancreatitis (15–21). Overall, it is not known whether substance use affects islet histopathology, including insulitis and insulin positivity; and the extent which substance use affects exocrine pancreatic histopathology in nPOD donors. In order to inform sample selection and study design of future research utilizing these valuable samples, we sought to determine the influence of alcohol, marijuana, tobacco, or illicit substance use on overall pancreas histopathology. We hypothesized that substance use would increase the odds of pancreatitis and chronic exocrine changes similarly in donors with and without type 1 diabetes but would not impact islet histopathology.

## 2 Materials and Methods

### 2.1 Donor Information

All nPOD type 1 diabetes and no-diabetes control organ donor tissue samples and records available up to November 2018 were reviewed for inclusion (n=304). Only donors ≥12 years of age were included (n=250). One type 1 diabetes donor had no information regarding age or duration of diabetes, five type 1 diabetes and control donors were missing substance use history, and two were missing pancreatic tissue and were excluded. Hence, 131 type 1 diabetes and 111 control pancreata were included in the final analysis (**Table 1**). All procedures were conducted in compliance with the United Network for Organ Sharing (UNOS) and the University of Florida Institutional Review Board requirements, based upon federal guidelines.

**Table 1:** Demographics and substance use patterns.

	Type 1 Diabetes (n = 131)	Controls (n = 111)
**Overall, n (%)**	131 (54.1%)	111 (45.9%)
**Male, n (%)**	71 (54.2%)	73 (65.8%)
**Race/Ethnicity, n (%)**		
Caucasian	102 (77.9%)	81 (73.0%)
African American	17 (13.0%)	19 (17.1%)
Hispanic/Latino	11 (8.4%)	11 (9.9%)
American Indian/Alaska Native	1 (0.8%)	–
**Mean Age at Death ± SD, years [range]**	31.2 ± 16.0 [12.0, 89.0]	28.2 ± 14.9 [12.0, 75.0]
**BMI At Death ± SD, kg/m^2^ [range]**	25.0 ± 4.6 [16.5, 42.5]	25.1 ± 5.4 [14.9, 38.8]
**Diabetes Duration ± SD, years [range]**	17.4 ± 16.8 [0.0, 84.0]	–
**Substance Use History, n (%)**		
Any Substance Use	87 (66.4%)	71 (64.0%)
Alcohol Use	71 (54.2%)	60 (54.1%)
Illicit Substance Use	28 (21.4%)	29 (26.1%)
Cocaine Use	20 (15.3%)	14 (12.6%)
Marijuana Use	40 (30.5%)	41 (36.9%)
Tobacco Use	56 (42.8%)	53 (47.8%)

### 2.2 Histology

Human pancreata were processed for fixed paraffin samples by the nPOD Organ Procurement and Pathology Core (22). From two blocks of each available region (head, body, tail), 4 µm paraffin sections were placed on a Superfrost Plus slide. Formalin fixed paraffin embedded (FFPE) pancreatic sections were stained with H&E and immunocytochemistry (IHC) according to standard protocols (23). Slides were scanned with an Aperio CS whole slide digital scanner at 20x and images were reviewed from the nPOD online pathology database (https://www.jdrfnpod.org/for-investigators/online-pathology-information/) for histopathology (24). Specifically, we evaluated insulin positivity, insulitis (≥6 CD3+ cells adjacent/within the islet in ≥3 islets/section) (25), amyloid staining, acute (acinar cell loss, polymorphonuclear cell infiltrate, fat necrosis, and hemorrhage) and chronic (acinar cell loss, mononuclear cell infiltrate, and fibrosis) pancreatitis, as well as chronic exocrine changes (acinar atrophy, fibrosis, fatty infiltration, or periductal fibrosis).

### 2.3 Data Collection and Management

Deidentified terminal hospital records were reviewed along with the deidentified Uniform Donor Risk Assessment Interview (UDRAI) wherein relatives were asked about the donor’s illicit substance, marijuana, smoking, and alcohol history (https://www.aatb.org/standards/uniform-drai). Illicit substance use was defined as any illicit substance other than marijuana (including amphetamines, barbiturates, bath salts, benzodiazepines, synthetic cannabinoids, cocaine, inhalants, LSD, MDMA, opioids, and PCP). In our analysis, subjects were considered to use the substance if they had any reported history of use. Due to the nature of the UDRAI and terminal hospital record reporting, the extent, timing, and duration of use of the substance of interest was not able to be reliably ascertained for all subjects and thus was not included in our analysis. nPOD donor data and histologic findings were recorded in REDCap, a web-based research tool designed for secure and rigorous data collection.

### 2.4 Statistical Analysis

Data were analyzed and graphed using SAS 9.4 (Cary, NC), and graphics produced using GraphPadPrism 8. Data are presented as mean ± standard deviation (SD) unless otherwise noted.

#### 2.4.1 Histopathologic Changes Compared to Substance Use

Pearson’s χ^2^ and t-tests were performed for categorical and continuous data, respectively, to compare demographics including sex, race/ethnicity, BMI at death, and substance use rates between type 1 diabetes and control donors. Odds ratios (OR) for rate of any histologic change within each substance use group were calculated for the overall population and within type 1 diabetes and control groups, both unadjusted and adjusted for age at death. In some instances, due to limitations of the data, these are not reported due to lack of model convergence, indicating the model itself is a poor fit and would not produce a reliable estimate. OR were calculated for the rate of individual histologic findings in substances of interest within the overall group and within type 1 diabetes and control populations adjusted for age at death. These included insulitis, insulin positivity, amyloid positivity, acute pancreatitis, chronic pancreatitis, and chronic exocrine changes within alcohol and cocaine users; and insulin positivity and insulitis within marijuana users. Associations were chosen because of suspected clinical association to limit the number of direct hypothesis testing.

#### 2.4.2 Histopathologic Changes in Type 1 Diabetes *Versus* Control Donors

Because it was noted descriptively that many donors with type 1 diabetes had histopathological evidence of acute and chronic pancreatitis along with other chronic exocrine changes, a secondary analysis was justified. Pearson’s χ^2^ was used to compare histologic changes in donors with type 1 diabetes versus controls before and after excluding donors with suspected DKA (n=75 donors with type 1 diabetes after exclusion) because of the known association of DKA with acute pancreatitis (26). Subjects were coded with probable DKA from terminal hospital record diagnoses or indicative laboratory values (pH <7.3, serum bicarbonate <15mEq/L, blood glucose >200mg/dL, and where available, evidence of serum or urinary ketosis).

## 3 Results

Demographics and descriptive findings are presented in **Table 1**. The average age ( ± SD) at time of organ donation was 29.8 (± 15.5) years with an average diabetes duration of 17.4 ( ± 16.8) years for those with type 1 diabetes. BMI, sex, and race/ethnicity were similar between groups. Rates of substance use were high and similar between controls (n=71, 64.0%) and individuals with type 1 diabetes (n=87, 66.4%).

### 3.1 Odds of Histopathologic Changes by Type of Substance Use

**Figure 1:**
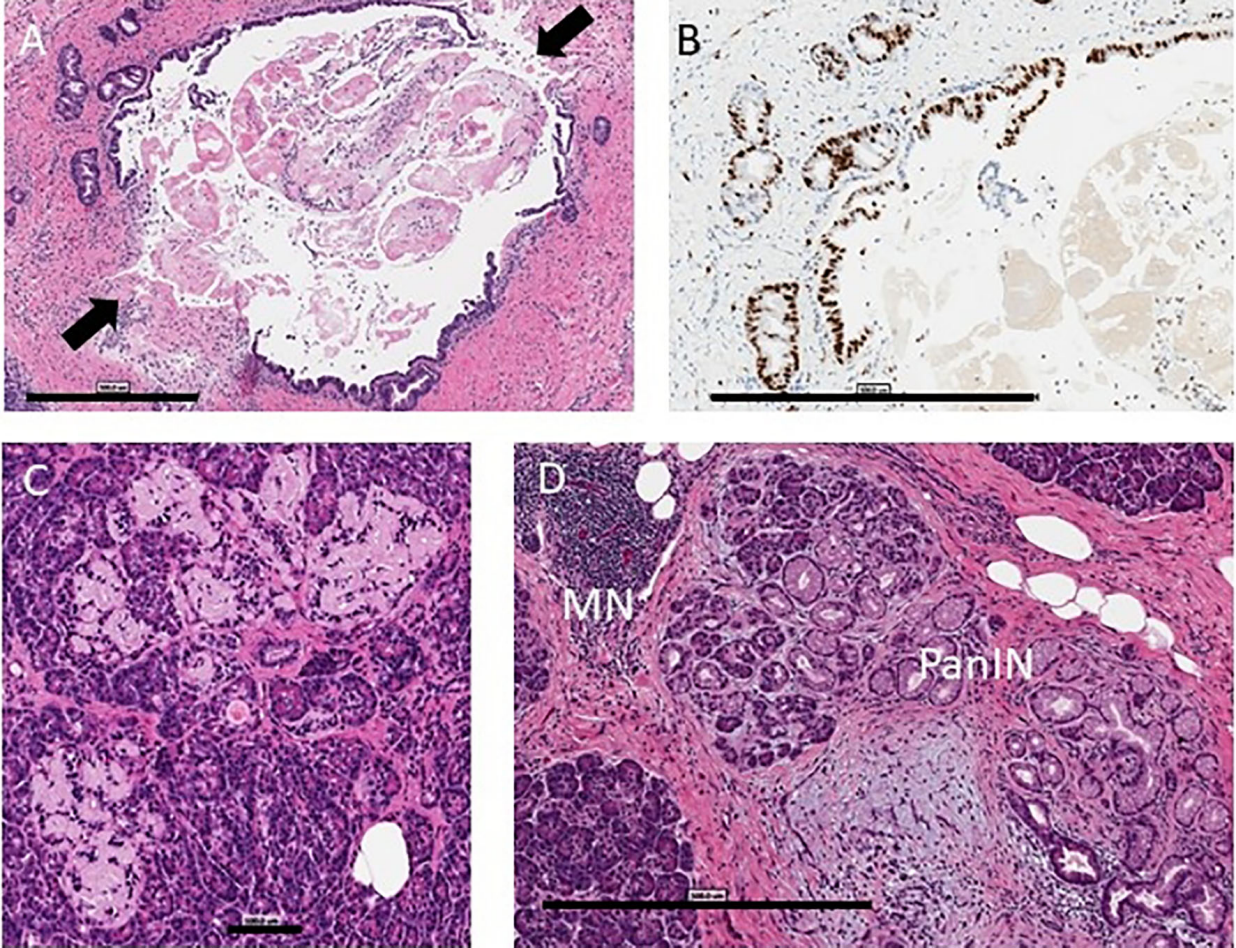
Pancreas histopathology. Representative images are shown from the nPOD Online Pathology database. The whole slide scans can be viewed with account request (https://www.jdrfnpod.org/for-investigators/password-request-form/). **(A)** Acute pancreatitis is shown by H&E staining in a 14.3 year old donor with type 1 diabetes for 8 years with no reported history of substance use (6089). The main pancreatic duct epithelium was breached at two points (arrows) on this image with intraluminal sludge observed by H&E staining. Scale bar: 500 µm. **(B)** The main duct and pancreatic duct glands showed very high epithelial Ki67+ cell numbers by IHC staining. Scale bar: 500 µm. **(C)** Islet amyloidosis in three islets is shown by H&E staining in a 24 year old donor with type 1 diabetes for 2 years who had a positive history of alcohol use and a positive blood ethanol level at the time of organ procurement (257 mg/dL) (6367). Scale bar: 100 µm. **(D)** Chronic pancreatitis is shown by H&E staining in a 30 year old donor with type 1 diabetes for 23 years (6266) with a positive history of tobacco use with mononuclear infiltration (MN), lobular fibrosis, and ductal dysplasia with pancreatic intraepithelial neoplasia (PanIN). Scale bar: 500 µm.

Alcohol, tobacco, marijuana, and illicit substance use did not increase the odds of having histopathologic changes as a group. When assessing individual histologic changes, substance use did not affect insulitis or insulin positivity. However, within type 1 diabetes donors, islet amyloid deposition was seen more frequently in users of alcohol (adjusted OR 9.96 [1.22, 81.29]) (**Figure 1C**), and chronic exocrine changes were more common in users of cocaine (adjusted OR 8.86 [1.13, 69.31]). Acute pancreatitis was seen more often within type 1 diabetes donors who used alcohol (adjusted OR 3.2 [1.06, 9.63]), but this relationship was no longer present after excluding donors with DKA.

### 3.2 Rates of Histopathologic Changes in Type 1 Diabetes *Versus* Control Donors

**Figure 2:**
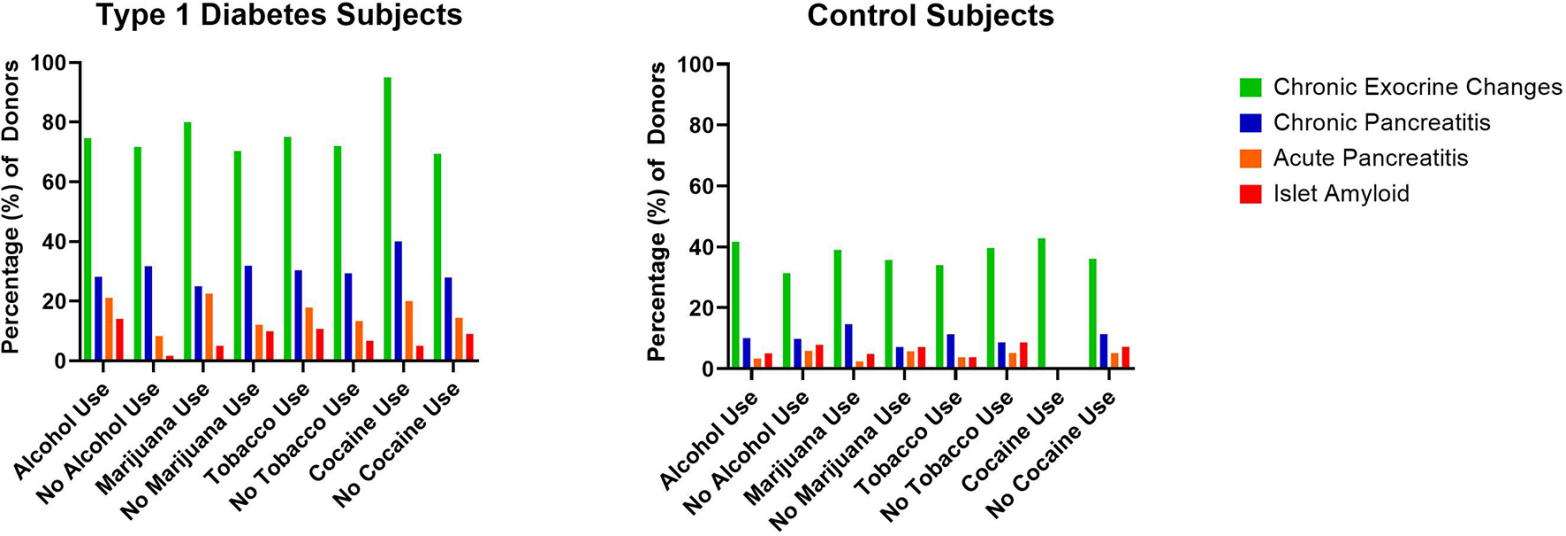
Percentage of type 1 diabetes (n=131) and control (n=111) pancreata from the Network for Pancreatic Organ donors with Diabetes (nPOD) with reported substance versus no substance use and associated histopathologic changes.

Histopathological evidence of chronic pancreatitis (29.8% versus 9.9%, p=0.0001) and chronic exocrine changes (73.3% versus 36.9%, p<0.0001), including acinar and periductal fibrosis and acinar atrophy, were present more frequently in type 1 diabetes versus control donors (**Table 2** and **Figures 1**, **2**). Histopathological evidence of acute pancreatitis was more often observed in donors with type 1 diabetes (15.3%) versus controls (4.5%, p=0.0061); however, this effect was no longer seen after excluding donors with DKA (8% versus 4.5%, p=0.3215) (**Table 2** and **Figure 1**).

**Table 2:** Pancreatic histopathologic changes.

Pancreatic Histopathology, n (%)	Type 1 Diabetes (n = 131)	Controls (n = 111)
Insulitis	37 (28.2%)	–
Insulin Presence	57 (43.5%)	111 (100.0%)
Islet Amyloid Present	11 (8.4%)	7 (6.3%)
Chronic Pancreatitis^a^	39 (29.8%)*	11 (9.9%)*
Acute Pancreatitis^a^	20 (15.3%)	5 (4.5%)
Chronic Exocrine Changes^a^	96 (73.3%)*	41 (36.9%)*
* Acinar Atrophy*	90 (68.7%)*	19 (17.1%)*
* Acinar Fibrosis*	55 (42.0%)*	14 (12.6%)*
* Fatty Infiltrate*	19 (14.5%)	31 (27.9%)
* Periductal Fibrosis*	17 (13.0%)*	3 (2.7%)*

a Includes all type 1 diabetes subjects from the original analysis (n=131). When type 1 diabetes donors with DKA were excluded (remaining n=75), percentages of type 1 diabetes subjects with findings were similar for chronic pancreatitis (36.0% versus 29.8%) and chronic exocrine changes (78.7% versus 73.3%) but lower for acute pancreatitis (6.0% versus 15.3%).

*Statistically significantly different (p < 0.01) between type 1 diabetes and control groups both when excluding and including type 1 diabetes donors with DKA.

## 4 Discussion

### 4.1 Statement of Principle Findings

We assessed the impact of a history of substance use on pancreatic histopathology in type 1 diabetes and control organ donors in the nPOD cohort. We observed a high but congruent rate of substance use in these two groups, similar to lifetime substance use patterns from a general population national survey (27). Importantly for future study of islet histopathology, insulitis and insulin positivity were not impacted by a history of substance use. However, alcohol and cocaine use did increase the odds of exocrine pathology and islet amyloid deposition within type 1 diabetes donors. Substance use impacted the pancreata of donors with type 1 diabetes more than controls. Interestingly, regardless of substance use history, our study found higher rates of exocrine histopathology including pancreatitis in individuals with type 1 diabetes versus controls; type 1 diabetes was a stronger risk factor than substance use for these changes.

### 4.2 Strengths and Weaknesses of the Study

Our study is the first to assess the effect of substance use on histopathologic findings in the nPOD biobank. Strengths of our study include the large number of samples examined and analysis of both type 1 diabetes and control donors. Despite our large sample size, the low prevalence of histopathologic changes in control donors and use of particular substances resulted in wide variation of odds. Another limitation is our reliance on reported substance use data in the medical chart and UDRAI. Due to the nature of this reporting, a more nuanced analysis assessing the impact of the timing, duration, and extent of substance use was not able to be performed. Unfortunately, only a very small subset of donors had serum or urine drug levels available, and these values would not capture all donors with history of substance use, since levels are cross-sectional and typically obtained only at the time of presentation to the healthcare facility.

### 4.3 Meaning of the Study

When assessing individual histologic changes in relation to use of specific substances, islet amyloid was more commonly observed in type 1 diabetes donors who used alcohol, a finding worth future study as increased levels of islet amyloid polypeptide have also been found in the setting of chronic alcoholic pancreatitis (28). Possible mechanisms for this phenomenon include glucokinase downregulation, fibroblast growth factor 21 (FGF21) resistance or deficiency, and changes in the pancreatic microcirculation in the setting of alcohol consumption which should be further explored (29–33). Type 1 diabetes subjects who used alcohol also had higher rates of acute pancreatitis, but this effect may have been driven by the presence of DKA or alternatively through previously described mechanisms of alcoholic pancreatitis (34). Chronic exocrine changes were more commonly seen in type 1 diabetes donors who used cocaine, which could potentially be explained by cocaine’s vasoconstrictive and atherosclerotic properties and is corroborated by previous findings of reduced exocrine pancreatic function in the setting of cocaine use (12, 35, 36). However, current versus past cocaine use was not able to be elucidated. Exocrine pathology including chronic pancreatitis, acinar and periductal fibrosis, and acinar atrophy was much more likely to occur in donors with type 1 diabetes, regardless of substance use history. Researchers should consider our findings when designing studies of the exocrine pancreas and/or islet amyloid in nPOD pancreata.

### 4.4 Unanswered Questions

Ongoing studies of the role of the exocrine pancreas in type 1 diabetes pathogenesis should continue to reveal pathways explaining these observed associations. Targeted prospective mechanistic studies evaluating the impact of cocaine and alcohol use on exocrine pancreatic function and islet amyloid polypeptide secretion are also needed. Assessment of laboratory markers of pancreatic function in the setting of cocaine and alcohol use could further the clinical implications of our findings. Validation in other cohorts, such as non-diabetic organ donors with islet autoantibodies, should be performed to further investigate the potential relationships between alcohol and cocaine use and pancreas histopathology within populations with and at-risk for type 1 diabetes. As the field of exocrine pancreas research within type 1 diabetes grows, the higher rates of pancreatitis and exocrine changes observed in this population should also be explored.

## Data Availability Statement

The datasets presented in this study can be found in online repositories. The names of the repository/repositories and accession number(s) can be found below: nPOD DataShare: npoddatashare.coh.org nPOD Online Pathology Database: https://aperioeslide.ahc.ufl.edu/Login.php.

## Author Contributions

DS and MA conceived of the study. BB and LJ designed the study and acquired, interpreted, and managed the data. BB drafted the manuscript. MC-T acquired and interpreted data. SF and MG managed, analyzed, and interpreted data. LJ is the guarantor of this work and had full access to all the data in the study and takes responsibility for the integrity of the data and accuracy of the data analysis. All authors were involved in editing the manuscript and had final approval of the submitted and published versions.

## Funding

This research was performed with the support of nPOD (nPOD; RRID : SCR_014641), a collaborative type 1 diabetes project sponsored by JDRF (nPOD: 5-SRA-2018-557-Q-R) and The Leona M. & Harry B. Helmsley Charitable Trust (Grant#2018PG-T1D053) with REDCap support from the National Center for Advancing Translational Sciences (NCATS grant UL1 TR000064). Additional support was provided by the National Institutes of Health (NIH) R01DK123329, U01DK127392, and R01DK122160 to MC-T; R01DK123292 and UC4DK108132 to MA; R01DK120357 to DS; and BB receives NCATS support through grant number KL2TR001429. The content and views expressed are the responsibility of the authors and do not necessarily reflect the official view of nPOD. Organ Procurement Organizations partnering with nPOD are listed at http://www.jdrfnpod.org/for-partners/npod-partners/. The study sponsor had no role in the design or interpretation of the study.

## Author Disclaimer

The content of this manuscript is solely the responsibility of the authors and does not necessarily represent the official views of the NIH.

## Conflict of Interest

The authors declare that the research was conducted in the absence of any commercial or financial relationships that could be construed as a potential conflict of interest.

## Publisher’s Note

All claims expressed in this article are solely those of the authors and do not necessarily represent those of their affiliated organizations, or those of the publisher, the editors and the reviewers. Any product that may be evaluated in this article, or claim that may be made by its manufacturer, is not guaranteed or endorsed by the publisher.

## Abbreviations

nPOD, Network for Pancreatic Organ donors with Diabetes; DKA, diabetic ketoacidosis; UDRAI, Uniform Donor Risk Assessment Interview; UNOS, United Network for Organ Sharing.
